# Reconstructed hyperspectral imaging for *in-situ* nutrient prediction in pine needles

**DOI:** 10.3389/fpls.2025.1630758

**Published:** 2025-08-11

**Authors:** Yuanhang Li, Jun Du, Chuangjie Zeng, Yongshan Wu, Junxian Chen, Teng Long, Yongbing Long, Yubin Lan, Xiaoliang Che, Tianyi Liu, Jing Zhao

**Affiliations:** ^1^ College of Electronic Engineering (College of Artificial Intelligence), South China Agricultural University, Guangzhou, China; ^2^ National Center for International Collaboration Research on Precision Agricultural Aviation Pesticides Spraying Technology, Guangzhou, China; ^3^ College of Forestry and Landscape Architecture, South China Agricultural University, Guangzhou, China

**Keywords:** hyperspectral image reconstruction, deep learning models, machine learning regression, *in situ* prediction of pine needle nutrients, precision forestry

## Abstract

**Introduction:**

Hyperspectral imaging (HSI) is a powerful, non-destructive technology that enables precise analysis of plant nutrient content, which can enhance forestry productivity and quality. However, its high cost and complexity hinder practical field applications.

**Methods:**

To overcome these limitations, we propose a deep-learning-based method to reconstruct hyperspectral images from RGB inputs for in situ needle nutrient prediction. The model reconstructs hyperspectral images with a spectral range of 400–1000 nm (3.4 nm resolution) and spatial resolution of 768×768. Nutrient prediction is performed using spectral data combined with competitive adaptive reweighted sampling (CARS) and partial least squares regression (PLSR).

**Results:**

The reconstructed hyperspectral images enabled accurate prediction of needle nitrogen, phosphorus, and potassium content, with coefficients of determination (R²) of 0.8523, 0.7022, and 0.8087, respectively. These results are comparable to those obtained using original hyperspectral data.

**Discussion:**

The proposed approach reduces the cost and complexity of traditional HSI systems while maintaining high prediction accuracy. It facilitates efficient in situ nutrient detection and offers a promising tool for sustainable forestry development.

## 1 Introduction

China is one of the countries with the widest pine tree planting area in the world. Many different types of pine trees have been widely planted in various parts of China, which are not only used for wood production and building materials, but also widely used in fields such as papermaking, chemical industry, and medicine (Yang et al., 2015; Leggate et al., 2020). Nutrient analysis of pine trees is crucial for understanding their growth environment, growth status, and wood quality (Yuan et al., 2015). By detecting the nutritional components of pine trees, their health status, growth rate, and soil quality in the environment can be evaluated, which helps to develop appropriate maintenance measures, improve the growth efficiency and wood quality of pine trees.

Hyperspectral imaging (HSI) is an advanced technology that acquires hundreds of narrow spectral bands at each spatial location, yielding a rich hyperspectral cube that integrates both spectral and spatial information. It enables qualitative and quantitative analysis of chemical composition, material quality, and other properties, making it a valuable tool for agricultural and forestry monitoring. Owing to its high spectral resolution and non-destructive nature, HSI is particularly well-suited for real-time and precision agriculture, allowing comprehensive assessments of crop growth and nutritional status (Fu et al., 2021; Wang et al., 2021; Zhao et al., 2020; Sethy et al., 2022).

Relying on these advantages, numerous studies in recent years have applied hyperspectral imaging (HSI) across a range of agricultural and forestry scenarios. This approach has demonstrated substantial application value; for example, in agriculture, He et al. improved leaf nitrogen estimation in winter wheat by employing multi-angle hyperspectral data (He et al., 2016), Yu et al. developed a spectral transfer vegetation index for rapid and accurate nitrogen inversion (Yu et al., 2022), Meiyan et al. applied spectral decomposition methods to estimate leaf nitrogen under UAV conditions (Meiyan et al., 2023), and Lan et al. combined near-infrared HSI with leave-one-out partial least squares regression in a two-step approach to assess internal apple fruit quality (Lan et al., 2021). In forestry applications, Ni et al. introduced a weight-adjusted convolutional neural network to predict nitrogen content in Masson’s pine seedlings using near-infrared spectra (Ni et al., 2019), while Li et al. integrated near-infrared spectra with partial least squares regression to estimate nitrogen content in different tissues of Masson’s pine (Li et al., 2022). These findings underscore the growing role of HSI as a key technology in agricultural and forestry applications, particularly for nutrient detection (Guo et al., 2021).

Despite its numerous advantages, the application of hyperspectral imaging (HSI) in agriculture and forestry is constrained by several factors. First, HSI technology struggles to achieve an optimal balance between spectral and spatial resolution; typically, higher spectral resolution comes at the expense of lower spatial resolution, and vice versa (Zhang et al., 2022). Second, the time-intensive nature of data collection and processing limits the real-time application of HSI (Arad et al., 2020). Additionally, the large volume of raw hyperspectral data hampers efficient transmission and storage. Furthermore, the high cost of hyperspectral cameras and related hardware restricts their widespread use, particularly for organizations with limited budgets (Fang et al., 2018; Signoroni et al., 2019). These factors collectively underscore the need for cost reduction, sensor fusion, and data-sharing platforms to enable broader HSI applications (Lan et al., 2024).

Consequently, research has shifted toward acquiring reliable hyperspectral information at lower cost. At this point, a novel approach has been proposed to reconstruct multichannel hyperspectral images from conventional RGB images. RGB imaging is more accessible and cost-effective than hyperspectral imaging; however, a key challenge lies in effectively mapping the limited information in RGB images to the rich spectral information in hyperspectral data (Shi et al., 2018). Fortunately, deep learning techniques have made remarkable progress in computer vision, profoundly transforming fields such as image classification, object detection, and segmentation. This progress has extended to hyperspectral imaging, where deep learning methods are now applied to hyperspectral reconstruction and related tasks. The combination of deep learning and hyperspectral imaging holds great potential for more efficient and accurate spectral data analysis (Zhang et al., 2022).

In recent years, deep-learning-based hyperspectral reconstruction techniques have become increasingly mature. As reconstruction accuracy has improved, numerous studies have demonstrated promising results across various applications. However, limited research has addressed the use of hyperspectral reconstruction for estimating plant nutrient components or quality grades in forestry and agriculture; most applications have predominantly focused on food testing. Zhao et al. applied a hyperspectral convolutional neural network with residual connections (HSCNN-R) to assess the soluble solid content (SSC) of tomatoes using only 36 samples, yielding a regression R² of 0.51 (Zhao et al., 2020). Yang et al. employed a pre-trained MST++ model to invert rice physiological parameters, achieving an R² of 0.40 for soil and plant analyzer development (SPAD) values (Yang et al., 2024). Recently, Ahmed MT et al. used HSCNN-R for hyperspectral reconstruction to predict SSC in sweet potato varieties, obtaining a regression R² of 0.69 on the test set (Ahmed MT et al., 2024b). Furthermore, in a comparative study by the same team, HSCNN-D, MST++, and HRNET were evaluated for their ability to predict the dry matter content (DMC) of sweet potato (Ahmed MT et al., 2024c). Yi et al. applied MPRNet to reconstruct visible and near-infrared spectra to identify the geographical origin of beef, achieving high classification accuracies of 98.58% and 94.38% on the training and testing sets, respectively (Yi et al., 2024). These studies collectively highlight a novel opportunity to leverage hyperspectral reconstruction methods for estimating the nutrient content of plants.

In this study, we improved the existing network architecture by extending the hyperspectral reconstruction range from 400–700 nm (31 bands) to 400–1000 nm (176 bands), while simultaneously enhancing the spectral resolution from 10 nm to 3.4 nm. This expansion aligns with the spectral range and resolution requirements for accurately estimating the content of key nutrients in pine needles. Four state-of-the-art models were adopted and modified as baselines: MIRNet (Multi-Scale Residue Network), HRNet (Hierarchical Regression Network), MPRNet (Multi-Stage Progressive Network), and Restormer (Restoration Transformer).

MIRNet (Zamir et al., 2020), introduced at ECCV 2020, demonstrated strong performance in the NTIRE 2022 spectral reconstruction challenge, achieving a PSNR of 33.29 dB. HRNet (Zhao et al., 2020), presented at CVPR 2019, utilizes a hierarchical structure and achieved 26.89 dB in the same task. MPRNet (Zamir et al., 2021), presented at CVPR 2021, and Restormer (Zamir et al., 2022), presented at CVPR 2022, employ encoder-decoder and transformer-based architectures, respectively; both performed strongly in NTIRE 2022, yielding PSNRs of 33.50 dB and 33.40 dB.

After modifying these four baseline models, we evaluated and compared their reconstruction performance. Ultimately, MIRNet demonstrated the highest accuracy and was selected to reconstruct hyperspectral images of pine canopies from RGB inputs. Spectral features extracted from these reconstructed images were then used in regression models to predict pine needle nutrient content. The reconstructed spectra generated by MIRNet achieved strong coefficients of determination (R²p) on the test set — 0.8523 for nitrogen, 0.7022 for phosphorus, and 0.8087 for potassium — closely matching the results obtained from original hyperspectral data (0.9038, 0.6815, and 0.8370, respectively), thereby validating the effectiveness of the proposed approach.

## 2 Materials and methods

### 2.1 Dataset preparation

**Figure 1:**
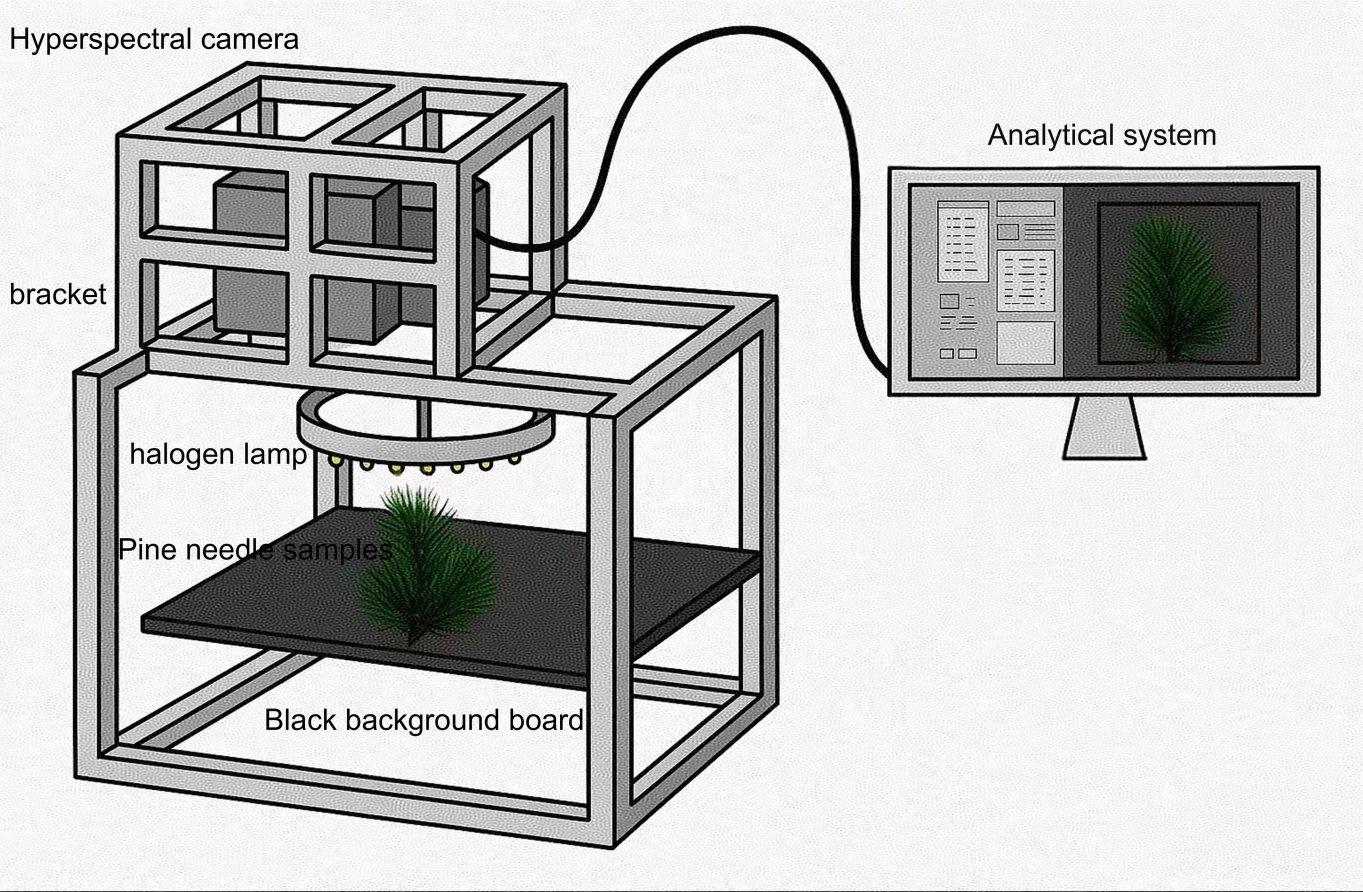
Hyperspectral imaging system.

This study aims to predict the nutritional composition of pine needles using a dataset comprised of canopy-level needle images from 282 trees of 1-year-old Pinus elliottii × Pinus caribaea hybrids. The experiment was conducted at the Hongling Seed Orchard Nursery in Taishan, Guangdong Province, China (22.164769°N, 112.822761°E). The seedlings were selected and potted by experts in soil composed of loess and a lightweight substrate in a 6:4 ratio, with a pot height of 16 cm. Among the 282 samples, 162 were used for hyperspectral reconstruction, while the remaining 120 were reserved for final nutrient prediction. Unlike many previous studies that rely on detached pine needles collected under laboratory conditions, this study preserved the structural integrity of the needles by conducting data collection *in situ* without any physical disruption (see **Figure 1**). Although the imaging was performed in a laboratory setting, the process maintained the original morphology of the samples. This approach enhances the ecological validity and generalizability of the resulting models. Consequently, models trained on this *in situ* dataset are more likely to accurately reflect real-world field conditions and maintain strong predictive performance in practical applications.

In this study, a visible and near-infrared hyperspectral camera (Gaifields Pro-V10, Dualix Spectrum Imaging Co.) was used to capture hyperspectral images of pine needle canopies under controlled laboratory conditions. The spectral range spanned 400–1000 nm and comprised 176 spectral bands. During image acquisition, illumination was provided by a halogen ring light with a photosynthetic photon flux density (PPFD) ranging from 88 to 95 µmol/(m²·s) and a lighting uniformity of 7.6%.

To simulate natural daylight and enhance the model’s generalizability to field conditions, RGB images were generated from hyperspectral data using the CIE 1931 color matching functions and the CIE standard illuminant D65. D65, with a correlated color temperature of approximately 6500 K, closely approximates the spectral distribution of natural midday outdoor light, encompassing both direct sunlight and diffuse skylight components. These RGB images were then used as input features for the spectral reconstruction model. Although the data were collected indoors, applying the D65-based RGB transformation allowed us to approximate realistic field illumination conditions, thereby improving the robustness and applicability of the trained model in outdoor scenarios.

During training, hyperspectral images were evenly divided into two subsets: 88 spectral bands in the visible range (400–700 nm) and the rest in the near-infrared range (700–1000 nm), which were used as training targets. This division was motivated by two main factors. First, spectral reflectance patterns in the visible range exhibit greater variability than those in the near-infrared range; training the model separately on these two segments can help improve reconstruction accuracy. Second, hyperspectral images contain large amounts of spectral information, which can lead to high memory consumption and slow GPU computation during training. By splitting the data into two parts, we enable parallel training, thereby improving computational efficiency and accelerating the training process.

### 2.2 Hyperspectral image reconstruction

#### 2.2.1 Improvement of four reconstruction models

This study evaluated four models whose original architectures were designed to reconstruct a spectral range of 400–700 nm, encompassing only 31 bands. This range falls predominantly within the visible spectrum and fails to provide sufficient spectral resolution and generalization capability for accurately analyzing pine needle nutrients. Furthermore, the lack of near-infrared (NIR) information limits the models’s ability to predict nutrient content, as NIR signals are strongly related to plant health. Previous research by Rahim Azadnia et al. identified key spectral regions associated with nitrogen (510–540 nm, 670–690 nm, 910–920 nm, and 985–995 nm), phosphorus (575–585 nm, 620–635 nm, 670–685 nm, 685–700 nm, and 965–975 nm), and potassium (505–515 nm, 555–570 nm, 590–605 nm, 685–700 nm, 920–930 nm, and 950–965 nm) (Azadnia et al., 2023). Similarly, Di Lin et al. applied the continuous wavelet transform (CWT) to identify effective NIR spectral regions for estimating nutrients; their study revealed four key spectral bands for nitrogen within 770–875 nm, four for phosphorus within 730–858 nm, and five for potassium within 755–890 nm (Lin et al., 2024). Consequently, the ability to accurately reconstruct segments of the NIR spectrum is crucial for improving the precision and reliability of nutrient predictions.

To address these limitations, we implemented several key modifications. The number of spectral bands was increased from 31 to 176, and the reconstruction range was expanded to 400–1000 nm to encompass the NIR spectrum (750–1000 nm). Furthermore, we applied a segmented computing approach by separately reconstructing the visible (400–700 nm) and NIR (700–1000 nm) regions. This strategy improved reconstruction accuracy while effectively retaining the essential NIR signals for more reliable nutrient estimation.

**Figure 2:**
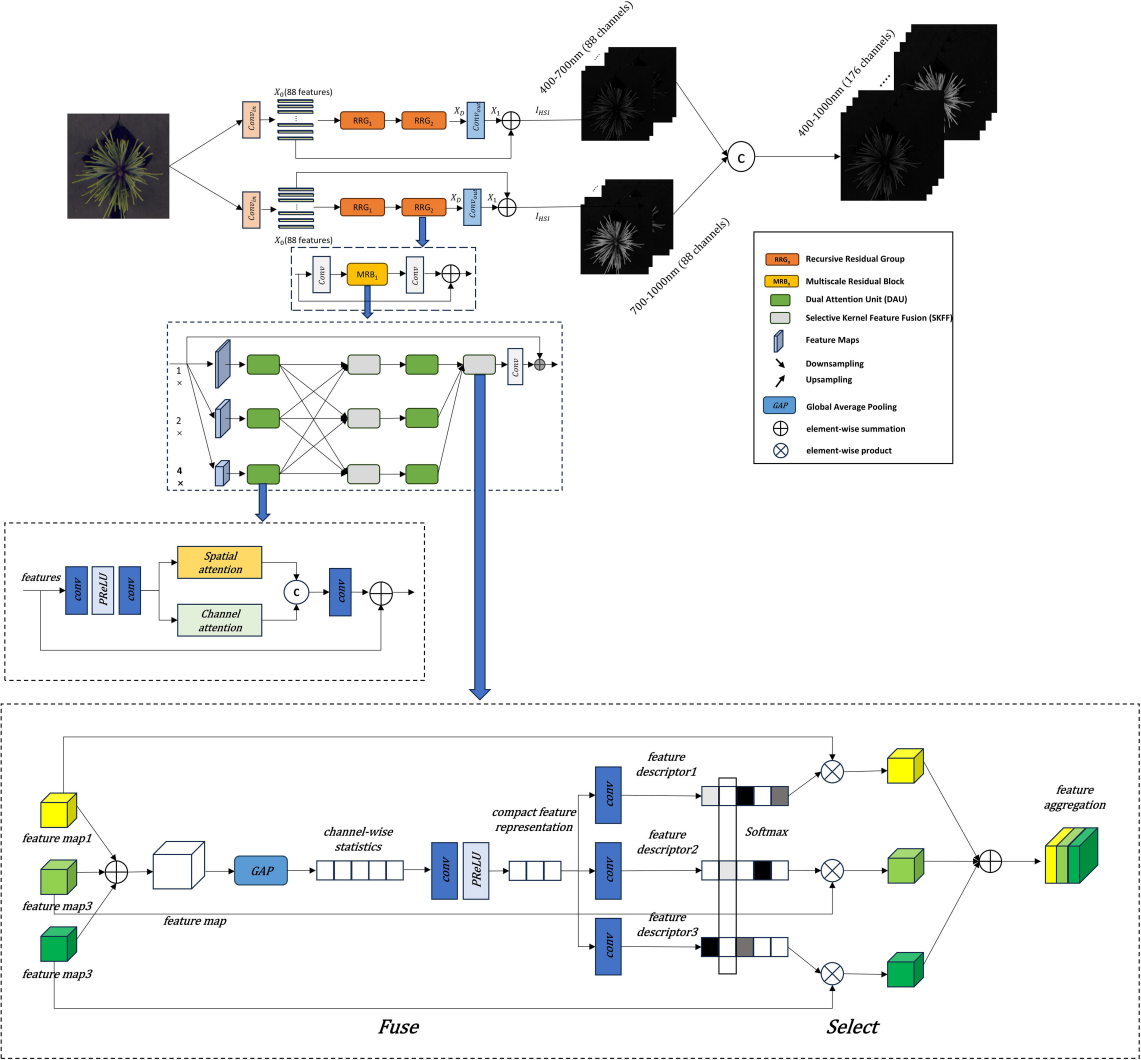
Improved MIRNet network architecture.

As shown in **Figure 2**, the improvements to the MIRNet model primarily involve several key modifications. First, at the input stage, the RGB image undergoes dimensional expansion to capture more detailed features. In this study, a 2D convolution is used to expand the three-channel RGB input to 88 channels. The expanded feature representation is then fed into subsequent network modules for further processing. One of the core strengths of the MIRNet model is its ability to maintain high-resolution representations while achieving high-quality image reconstruction. This is accomplished by integrating Multi-Resolution Residual Blocks (MRB), Selective Kernel Feature Fusion (SKFF), and Dual Attention Units (DAU). In the improved model, the number of Recursive Residual Groups (RRG) is set to 2, with each RRG containing one MRB module. Additionally, the number of upsampling and downsampling layers within the MRB module is set to 3. The final output consists of two 88-dimensional spectral components, which are merged to form the final 176-dimensional reconstructed hyperspectral cube.

**Figure 3:**
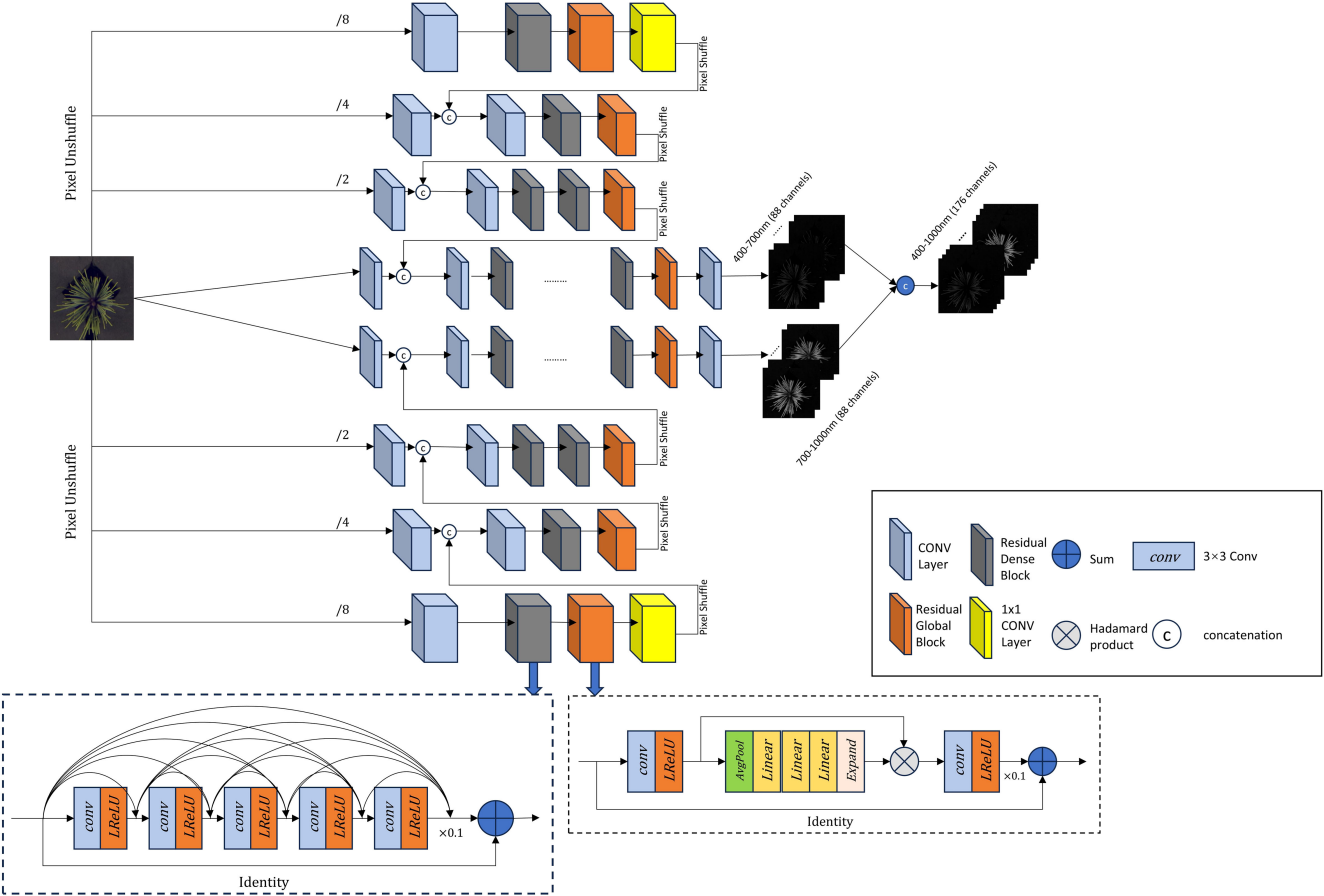
Improved HRNet network architecture.

As shown in **Figure 3**, the improved HRNet adopts a U-shaped structure. The three-channel RGB image is fed into an eight-layer hierarchical framework, where every two layers process different feature scales. The input RGB image is first expanded into an 88-dimensional feature cube. From the middle towards both ends, the channel resolution progressively increases while the spatial resolution decreases. At the middle of the model, the two central layers consist of two 2D convolutional modules, four Residual Dense Blocks, one Residual Global Block, and an additional 2D convolutional module. The remaining six layers follow the structure shown in the figure, each ending with a PixelShuffle operation. This operation rearranges the feature maps to match the shape of the preceding layer and connects them accordingly. This upsampling and downsampling structure preserves feature integrity, maintaining the original data distribution while ensuring adaptability. As a result, it enhances reconstruction accuracy while effectively capturing essential details.

**Figure 4:**
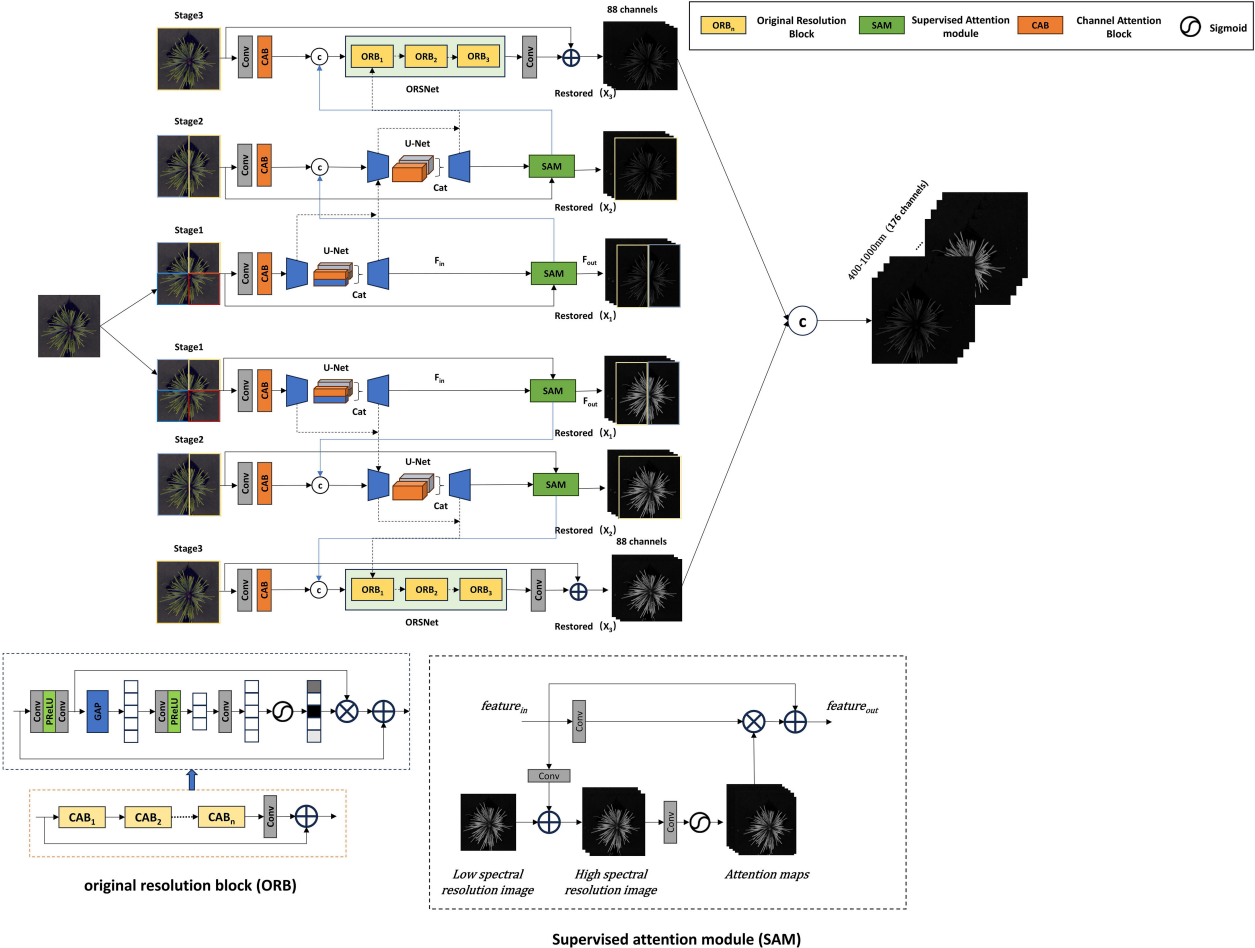
Improved MPRNet network architecture.

For the improvement of the MPRNet model, as shown in **Figure 4**, a process similar to MIRNet is adopted, where the three-channel RGB image is first expanded to 88 dimensions through an input convolution. Utilizing an encoder-decoder architecture, the reconstruction task is divided into multiple progressively optimized subtasks across two spectral ranges. In this study, the process is structured into three stages. In the first stage, the input image is divided into four parts. In the second and third stages, the network gradually expands the reconstruction range while learning reconstruction functions to capture multi-scale contextual information. In the third stage, the number of Original Resolution Blocks (ORB) is set to three. Finally, features from different scales are integrated to generate the final 176-dimensional reconstructed hyperspectral image. The key characteristics of this model include the use of a Supervised Attention Module (SAM) for progressive learning and the implementation of a Cross-Stage Feature Fusion (CSFF) mechanism to enhance feature propagation across stages, ensuring the extraction of more fine-grained spectral information.

**Figure 5:**
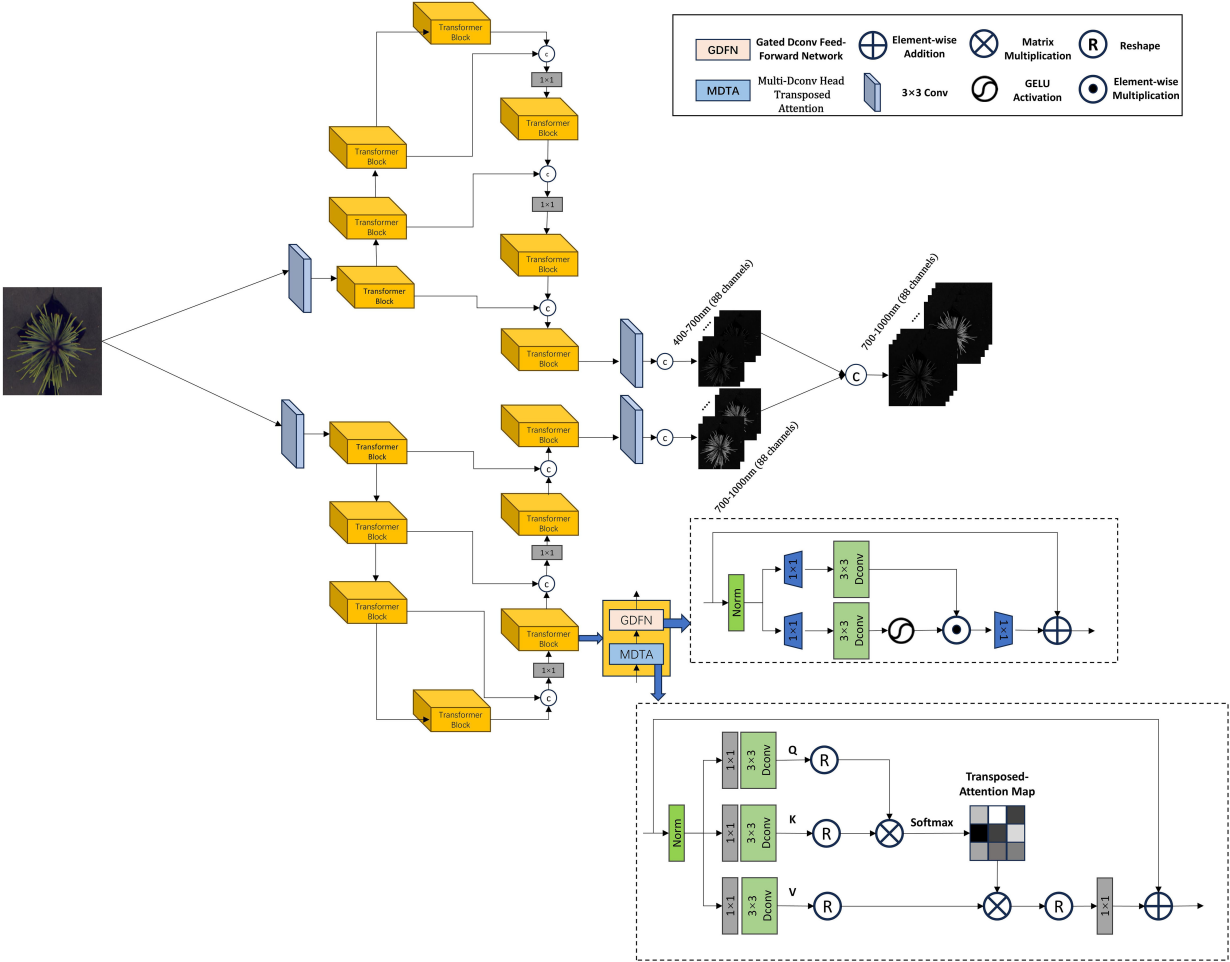
Improved Restormer network architecture.

In the improved Restormer model, as shown in **Figure 5**, the three-channel RGB image is first processed through an overlapped patch embedding layer, expanding its dimensions from 3 to 108. The transformed data is then fed into the Transformer blocks of the encoder, where the spatial resolution is enhanced while the number of channels is progressively reduced. In this study, the entire model is structured into two parallel branches, each consisting of a four-layer architecture. The number of Transformer blocks in these four layers is set to 2, 3, 3, and 4, respectively. At the end of each branch, the extracted features are passed into the decoder, which is also composed of Transformer blocks. The decoder refines the features while recovering high-resolution details. Finally, the features from both branches are merged to form the final 176-dimensional hyperspectral cube. The Restormer model employs a Multi-Dconv Head Transposed Attention mechanism within its Transformer blocks. This attention layer enhances feature representation by performing query-key interactions across channels. Additionally, Restormer introduces a Gated Feed-Forward Network (GFN), which selectively allows only informative features to propagate through the network, thereby improving the efficiency of information transmission.

#### 2.2.2 Quality evaluation

In the field of spectral image reconstruction, researchers typically use multiple image quality evaluation metrics to assess the quality of hyperspectral images reconstructed from RGB images. These indicators aim to quantify the differences between reconstructed hyperspectral images and ground truth hyperspectral images, and provide objective measures of the quality of spectral image reconstruction. In this study, Mean Relative Absolute Error (MRAE), Root Mean Square Error (RMSE), and Peak Signal to Noise Ratio (PSNR) were used as evaluation metrics. During the model training process, we are particularly concerned with the MRAE coefficients. The MRAE was used as the loss function to enhance resistance to outlier data and enable uniform evaluation of each band under varying lighting conditions (Shi et al., 2018), and the model will be trained until this metric converge. The model with the best performance during the training phase will be selected for the final hyperspectral reconstruction, and all three-evaluation metrics will be used in the evaluation process of the testing and validation dataset. Their definitions are shown in Equations 1-3. H and W represent the height and width of the image, i and j represent the coordinates of pixels in the height and width of the image, $I_{G}$ represents the ground truth spectral image, $I_{R}$ represents the reconstructed spectral image, peak is the scalar of the peaking signal value.


(1) $$\begin{array}{c} \text{MRAE}=\frac{1}{\text{H}\times \text{W}}\sum_{\text{i}=1}^{\text{H}}\sum_{\text{j}=1}^{\text{W}}\left(\frac{\left|{\text{I}}_{\text{R}}\left(\text{i},\text{j}\right)-{\text{I}}_{\text{G}}\left(\text{i},\text{j}\right)\right|}{{\text{I}}_{\text{G}}\left(\text{i},\text{j}\right)}\right) \end{array}$$

(2) $$\begin{array}{c} \;\text{RMSE}=\sqrt{\frac{1}{\text{H}\times \text{W}}\sum_{\text{i}=1}^{\text{H}}\sum_{\text{j}=1}^{\text{W}}{\left({\text{I}}_{\text{G}}\left(\text{i},\text{j}\right)-{\text{I}}_{\text{R}}\left(\text{i},\text{j}\right)\right)}^{2}} \end{array}$$

(3) $$\begin{array}{c} \;\text{PSNR}=10\text{lg}\left(\frac{\text{pea}{\text{k}}^{2}}{\text{RMS}{\text{E}}^{2}}\right) \end{array}$$

### 2.3 Nutritional component prediction

In this study, the processing of pine needle hyperspectral images first involved the extraction of regions of interest (ROIs) to suppress interference from the black background board and retain only the effective area of the pine needle. To achieve this, a threshold was applied to generate a mask, which was then used to extract spectral data for subsequent analysis. The average spectral reflectance of each channel was computed, yielding a one-dimensional array of 176 values. To minimize the effects of external conditions and instrument variability on spectral data, a thorough preprocessing pipeline was implemented. This pipeline included denoising, wavelength calibration, and spectral calibration to help ensure data accuracy and reliability and to enhance the precision of subsequent machine learning regression. Specifically, Multiple Scatter Correction (MSC) and First Order Differentiation (D1) were applied to improve spectral data quality and regression accuracy. To further reduce data dimensions and optimize model performance, the Competitive Adaptive Reweighted Sampling (CARS) algorithm was used to select representative spectral bands. CARS performs iterative sampling and PLS regression calculations to identify the most informative spectral variables, thereby improving the efficiency and robustness of data processing and model development.

In the regression stage of this study, samples were divided into training and testing sets at a ratio of 8:2, with the testing set reserved for final model evaluation. To predict nutrient content, we employed Partial Least Squares Regression (PLSR), a classical regression method that combines the dimensional reduction capabilities of Principal Component Analysis (PCA) with multivariate regression. PLSR identifies new components that maximize the covariance between independent and dependent variables, establishing a linear relationship between them. It is widely used in spectral analysis and nutrient modeling due to its ability to handle high-dimensional data and multicollinearity.

During training, ten-fold cross-validation was employed to enhance the model’s robustness and generalization capability. To comprehensively evaluate predictive performance, three key metrics were utilized: the prediction coefficient of determination (R²p), the root mean square error of prediction (RMSEP), and the relative prediction deviation (RPD), as defined in Equations 4-6. In these equations, $SS_{residual}$ denotes the sum of squared residuals—the squared differences between predicted and observed values—while $SS_{total}$ represents the total sum of squares, indicating the variability of the observed values around their mean. ${\hat{y}}_{i}$ and ${\hat{y}}_{i}$ represent the predicted and actual values, respectively. These metrics provide a direct assessment of the model’s generalization and predictive accuracy on unseen data. Specifically, a higher R²p, lower RMSEP, and larger RPD reflect improved predictive performance and greater practical utility. Therefore, these three metrics are used as the primary indicators for evaluating the final effectiveness of the model in this study.


(4) $$\begin{array}{c} {\text{R}}^{2}=1-\frac{\text{S}{\text{S}}_{\text{residual}}}{\text{S}{\text{S}}_{\text{total}}} \end{array}$$

(5) $$\begin{array}{c} \text{RMSE}=\sqrt{\frac{1}{\text{n}}\sum_{\text{i}=1}^{\text{n}}{\left({\hat{\text{y}}}_{\text{i}}-{\text{y}}_{\text{i}}\right)}^{2}} \end{array}$$

(6) $$\begin{array}{c} \text{RPD}=\frac{1}{\sqrt{\left(1-{\text{R}}^{2}\right)}} \end{array}$$

### 2.4 Computational environment

The computations in this study were conducted using the cloud computing platform Autodl. The remote server was supported by a 15v CPU Intel(R) Xeon(R) Platinum 8474C, 80GB of memory, and an RTX 4090D (24GB). During the training process, the number of training epochs was set to 200, with each epoch containing 1000 iterations, the batch size of the training set was 2, the learning rate was 1e-4, and the dimensions of the dataset used for training were 768×768. The patch size was set to 128 with a stride of 8. For adjusting the learning rate, the Adam optimizer was employed with parameters β_1_ = 0.9 and β_2_ = 0.999. The analysis was conducted using Python 3.8.19. The reconstruction model was trained using open-source Python packages, including Scikit-learn, PyTorch, and OpenCV-Python.

## 3 Results

### 3.1 Reconstruction performance of the models in this study

The initial step in training the reconstruction model involved processing the input RGB images and preparing the corresponding hyperspectral data as ground truth. As detailed in Section 2.1, two segments of hyperspectral data were used as the reference, with the matching RGB images serving as inputs to the model. The training dataset comprised 140 images, while the validation dataset included 22 images. Training continued until the loss convergence criterion was satisfied, after which the best-performing model was selected for further evaluation. **Table 1** summarizes the parameter count, computational cost, and validation performance of the four models across the 400–700 nm and 700–1000 nm spectral ranges.

**Table 1:** Reconstruction results.

Methods	Params(M)	FLOPS(G)	Band range	MRAE	RMSE	PSNR
MIRNet	20.18M	58.15 G	400-700nm	0.4424	0.0349	29.2153
			700-1000nm	0.3233	0.0531	25.6548
HRNet	59.45 M	77.79 G	400-700nm	0.4567	0.0357	29.0037
			700-1000nm	0.3282	0.0549	25.3349
MPRNet	29.16 M	320.45 G	400-700nm	0.4475	0.0354	29.0935
			700-1000nm	0.3342	0.0551	25.3228
Restormer	75.66 M	106.10 G	400-700nm	0.4668	0.0352	29.1454
			700-1000nm	0.3439	0.0560	25.1091

In our study, the MIRNet model demonstrated strong performance in hyperspectral reconstruction, particularly across both the 400–700 nm and 700–1000 nm ranges. For the 400–700 nm range, MIRNet achieved a minimum mean relative absolute error (MRAE) of 0.4424, a root mean square error (RMSE) of 0.0349, and a peak signal-to-noise ratio (PSNR) of 29.22 dB. For the 700–1000 nm range, the corresponding values were 0.3233, 0.0531, and 25.65 dB, respectively. Additionally, MIRNet exhibited the lowest computational cost among the tested models, requiring only 58.15 gigaflops and featuring the smallest model size of 20.18 million parameters. These attributes significantly improved the algorithm’s efficiency and supported the deployment of future embedded applications.

### 3.2 Comparison of the performance of existing methods

To more clearly demonstrate the superiority of our proposed model, this section reviews and compares the performance of several representative methods in hyperspectral reconstruction tasks in recent years. These methods, including HSCNN-D, HRNet, and MPRNet, serve as intuitive and compelling benchmarks against which we can evaluate and highlight the advantages of our approach. In a study by Md. Toukir Ahmed et al. investigating agricultural product quality through hyperspectral image reconstruction, the HSCNN-D model was used to reconstruct 204 spectral bands (400–1000nm) from RGB images of sweet potato. The model achieved a mean relative absolute error (MRAE) of 0.8601, a root mean square error (RMSE) of 0.0545, and a peak signal-to-noise ratio (PSNR) of 26.86 dB on the validation set. On the test set, the corresponding metrics were 0.7914, 0.0566, and 25.91 dB, respectively (Ahmed et al., 2024b). Furthermore, in another study by the same team focusing on chicken embryo mortality, the HRNet model demonstrated improved reconstruction performance, yielding an MRAE of 0.0936, RMSE of 0.0151, and PSNR of 36.79 on the validation set (Ahmed et al., 2024a). However, this study reconstructed only 10 spectral bands within the 520–903nm range, which likely contributed to its higher reconstruction accuracy due to reduced spectral complexity.

Notably, in a study by Weiguo Yi et al. on geographical origin identification of beef, the MPRNet model was applied to reconstruct hyperspectral images (400–1000nm, 125 bands) from RGB inputs. The model performed exceptionally well, yielding an MRAE of 0.177, RMSE of 0.0165, and PSNR of 35.76 (Yi et al., 2024). The high reconstruction accuracy in this study may be due to the large proportion of beef regions within each image and their relatively flat surface characteristics, which minimize depth of field effects. Additionally, the small number of spectral bands to be reconstructed further facilitated accurate reconstruction. Overall, existing methods all demonstrate a certain ability to reconstruct hyperspectral images and are applicable to a range of datasets and application scenarios. However, they struggle to balance reconstruction accuracy with spectral resolution. Our approach effectively overcomes this limitation, offering a more robust solution that achieves both high reconstruction accuracy and strong spectral fidelity.

### 3.3 Reconstructed spectral quality assessment

In the final prediction of nutrient components, spectral data within the 400–450 nm and 950–1000 nm ranges were deliberately excluded. This decision was based on two primary considerations: the high noise levels observed in these spectral regions and their limited relevance to the absorption peaks of the three essential nutrients—nitrogen, phosphorus, and potassium. Including such noisy and non-informative wavelengths could compromise prediction accuracy. To enhance both model efficiency and predictive performance, the spectral range of 450–950 nm was selected for nutrient content estimation. Following hyperspectral image reconstruction, spectral data within this range were extracted for subsequent regression analysis, and the quality of the reconstructed spectra was rigorously evaluated.

**Figure 6:**
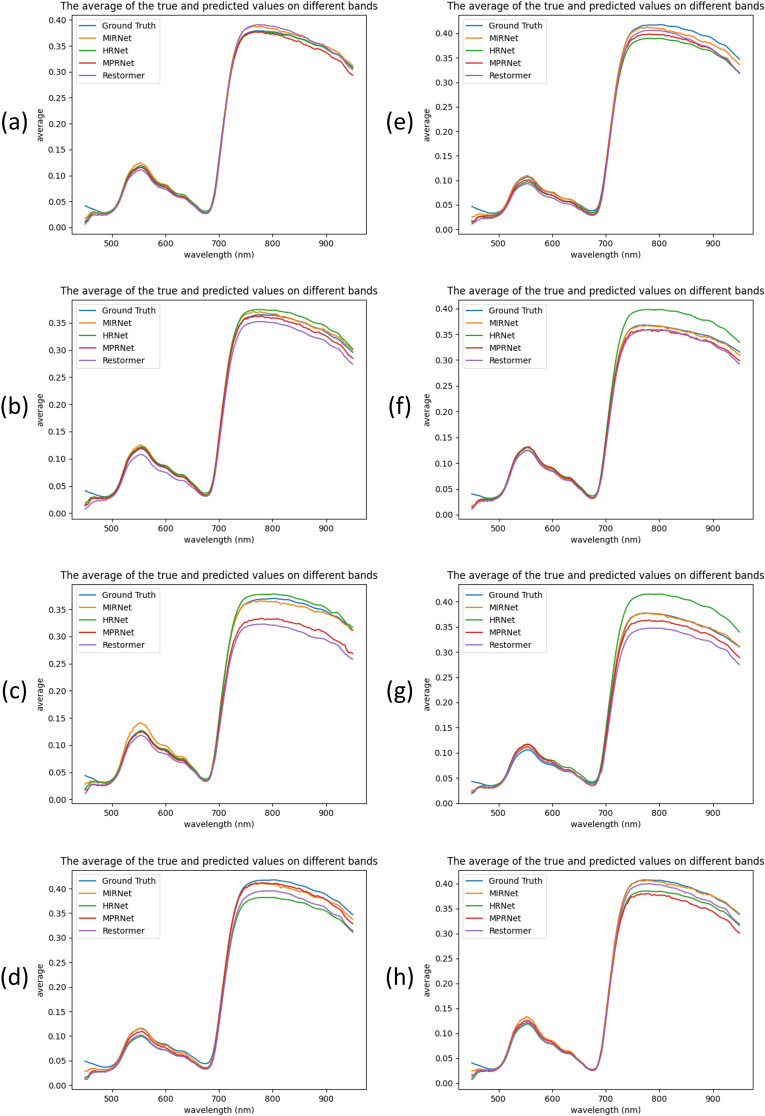
Comparison of the reconstructed spectra of four models and the ground truth spectra on eight samples. **(a–h)** represent eight different pine needle samples.

To assess model generalization, eight samples were randomly selected from the 120 test set samples, as shown in **Figure 6**. The results demonstrate that MIRNet and MPRNet generally provided the best spectral alignment with the ground truth, whereas Restormer and HRNet exhibited more pronounced reconstruction errors across specific spectral bands.

Specifically, MIRNet consistently showed superior spectral fitting across most samples, particularly in wavelengths beyond 700 nm. In contrast, MPRNet exhibited notable fluctuations in this range, especially in samples c and h. HRNet performed exceptionally well on sample c; however, for other samples—particularly f and g—it displayed considerable error fluctuations beyond 700 nm, indicating variable reconstruction stability. Restormer exhibited inconsistent performance across all eight samples, with marked discrepancies observed in samples c, d, and g.

It is also worth noting that in samples a, c, h, and h, MIRNet slightly overestimated the 550 nm peak relative to the ground truth. Nonetheless, in the context of full-spectrum reconstruction, MIRNet demonstrated the most stable and accurate performance, while utilizing the fewest parameters and the least computational resources among all models evaluated. This strong performance may be attributed to MIRNet’s selective kernel fusion mechanism, which effectively integrates features from different receptive fields and enhances the model’s ability to manage spatial and channel resolution challenges.

### 3.4 Visual quality assessment of the reconstructed images

**Figure 7:**
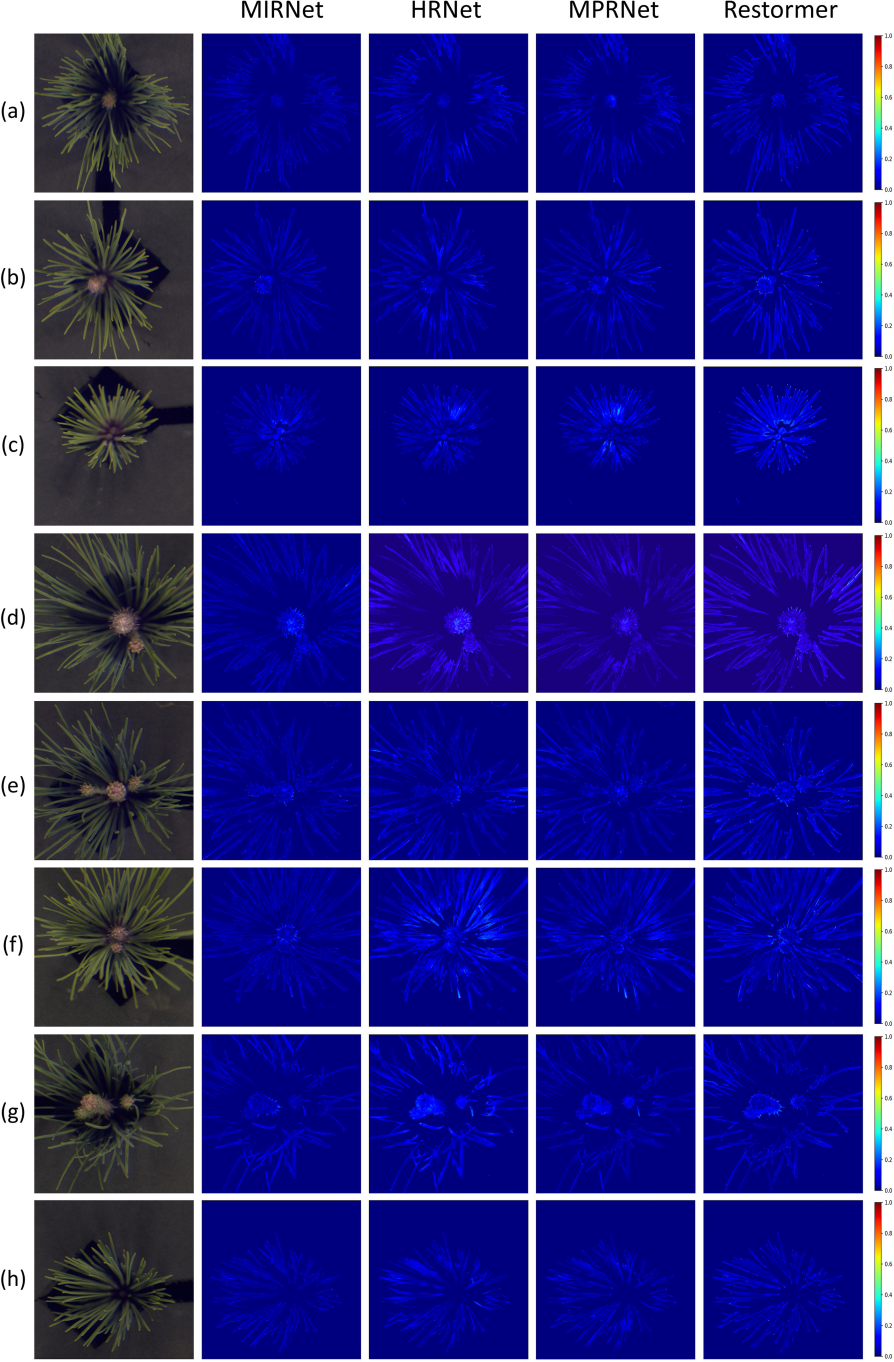
Error heatmaps of four networks based on the MRAE metric at 700 nm for eight samples. **(a–h)** correspond to the same eight pine needle samples as shown in **Figure 6**.

To more intuitively illustrate the performance of hyperspectral reconstruction, visual analysis was incorporated into the evaluation process. The Mean Relative Absolute Error (MRAE) index was first used to generate error heatmaps, showing the differences between the reconstructed spectra at 700 nm and the ground truth spectra for four representative samples, as shown in **Figure 7**. These heatmaps clearly depicted the spatial distribution of reconstruction errors across the pine needle regions for each model.

The visual analysis highlighted the superior performance of MIRNet, which exhibited minimal errors throughout the entire needle area. In comparison, MPRNet showed moderate errors, particularly in the needle regions of samples c and f. HRNet demonstrated more pronounced errors in samples d, f, and g, with a similar distribution pattern to MPRNet across other samples. Restormer performed comparably to MPRNet in sample f but exhibited substantial errors in other samples, especially sample c. Combined with the spectral curve analysis discussed earlier, these results suggest that Restormer struggles with reconstructing fine-grained regions in the sample data.

**Figure 8:**
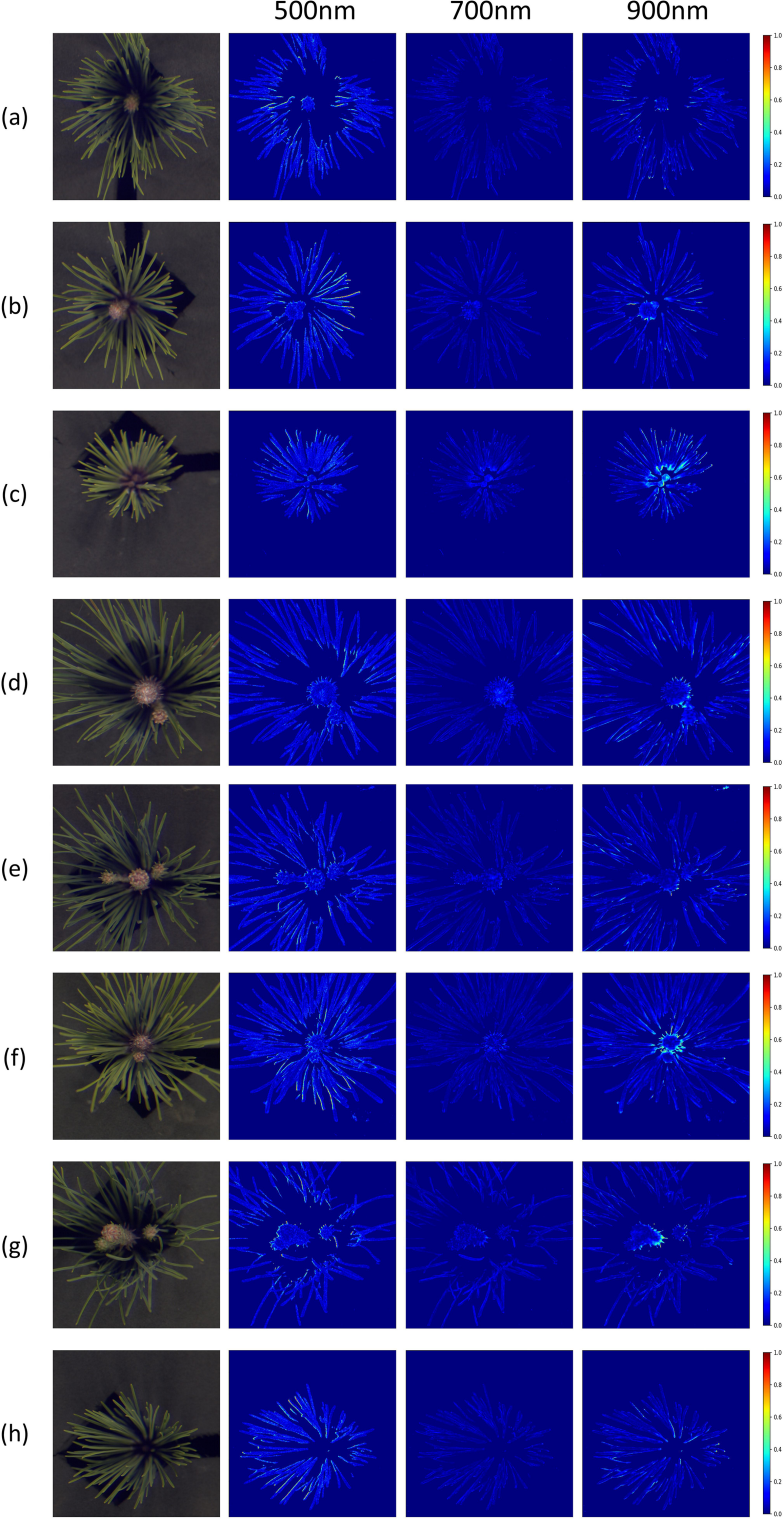
Error heatmaps generated by the MIRNet model using the MRAE metric at 500 nm, 700 nm, and 900 nm across eight pine needle samples. Subfigures **(a–h)** correspond to the same eight samples as in **Figure 6**.

Additionally, MRAE-based heatmaps were generated to compare the reconstruction accuracy of the best-performing MIRNet model against the ground truth at 500 nm, 700 nm, and 900 nm wavelengths, as illustrated in **Figure 8**. These heatmaps further revealed spatial variations in reconstruction quality across the pine needle structures. At 500 nm, significant errors were observed in the needle areas, likely due to surface smoothness and irregularities causing uneven light reflection and refraction, particularly in shadowed regions. At 900 nm, while the outer edges of the needles exhibited accurate reconstructions, the central regions showed larger errors. This is likely attributable to the limited depth of field during image acquisition, compounded by shadowing effects and strong near-infrared responses in dark areas not easily visible in the RGB spectrum. These findings emphasize the challenges of achieving uniform reconstruction across varying spatial and spectral characteristics.

### 3.5 Prediction results

In this section, the MIRNet model, which achieved the highest reconstruction performance, was employed for final regression prediction.

**Figure 9:**
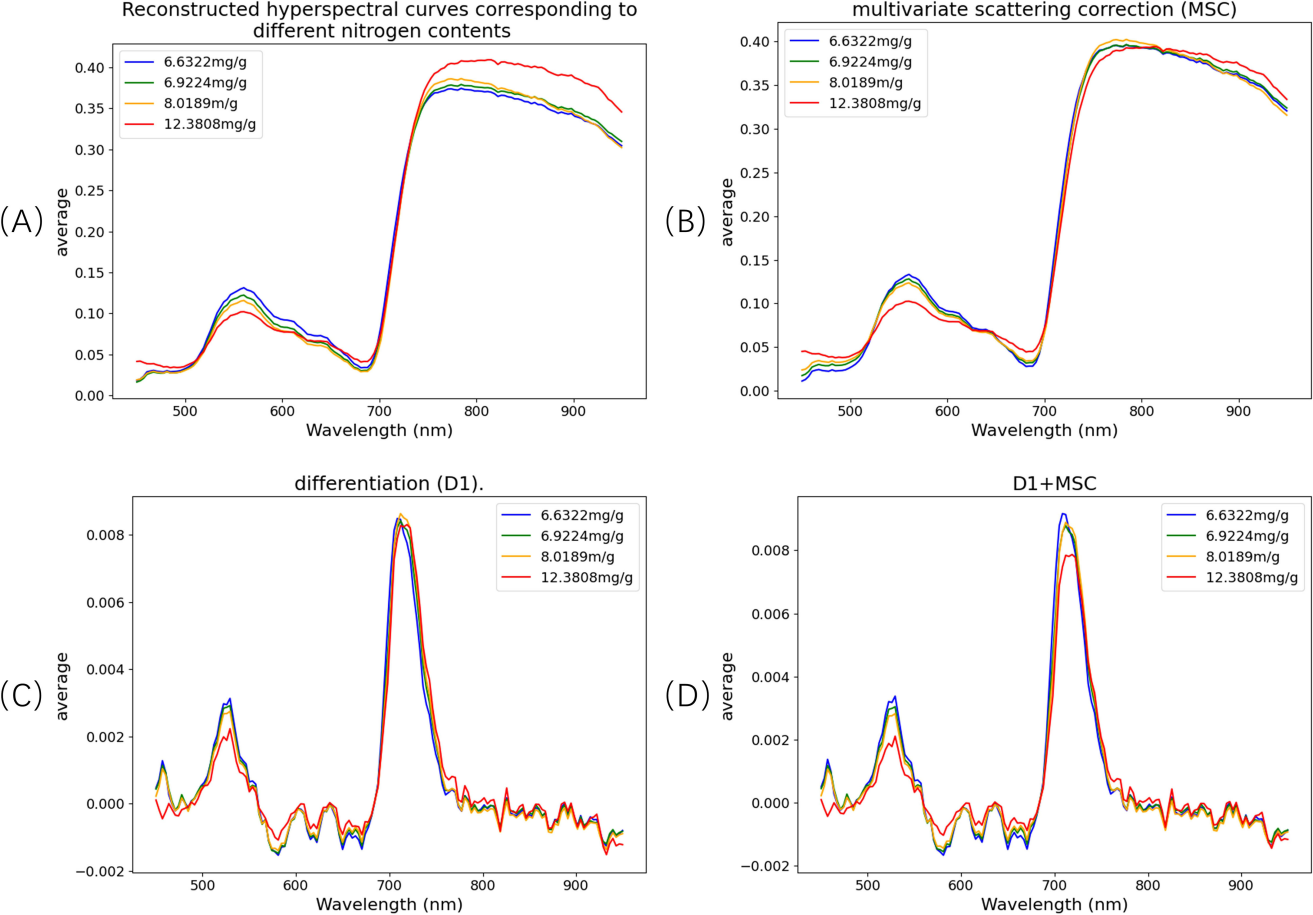
Spectral curves at different nitrogen concentrations and spectral curve after pre-processing. **(A)** Original spectrum; **(B)** multivariate scattering correction (MSC); **(C)** differentiation (D1); **(D)** D1+MSC.

**Figure 9** presents the hyperspectral reflectance curves of pine needle canopy samples with varying nitrogen concentrations across the 450–950 nm spectral range. The reflectance curves display distinct patterns corresponding to four nitrogen levels. Specifically, in the 450–600 nm region, reflectance decreases as nitrogen concentration increases. This trend is attributed to the positive correlation between nitrogen content and chlorophyll levels in plants. Chlorophyll strongly absorbs light in the 450–470 nm and 630–670 nm bands, leading to reduced reflectance and broader absorption features, particularly resulting in a diminished green peak around 550.8 nm.

These spectral variations underscore the sensitivity of hyperspectral signals to nutrient content and validate the potential of hyperspectral data for non-destructive estimation of nitrogen levels in pine canopies.

Regions of interest (ROIs) were extracted from the reconstructed spectral data, followed by spectral preprocessing. In this study, three preprocessing methods were applied: Multiplicative Scatter Correction (MSC), first derivative (D1), and a combination of D1 and MSC (D1+MSC). The original spectra and the results after applying these preprocessing techniques are illustrated in **Figure 9**.The MSC method enhances spectral bands with greater variability by computing the mean spectrum and correcting the original spectra through offset adjustments. This process effectively reduces spectral differences in the 800–987 nm range, corrects scattering effects, and improves the linearity of chemical information. The D1 method eliminates baseline drift and noise interference while enhancing spectral resolution and detail. The combined D1+MSC approach integrates the advantages of both methods, optimizing the signal-to-noise ratio and feature separability, thereby significantly improving the accuracy and reliability of multi-component detection in complex systems.

Following preprocessing, the Competitive Adaptive Reweighted Sampling (CARS) algorithm was employed to extract characteristic wavelengths, aiming to eliminate redundant bands and enhance predictive accuracy. These selected wavelengths serve as key indicators in spectral analysis. By optimizing the weight of data at these specific wavelengths, both spectral reconstruction and nutrient content prediction performance can be substantially improved. The extracted spectral features were then used to further evaluate the MIRNet model. Additionally, it was observed that the nutrient content values used as labels had a relatively narrow range, particularly for phosphorus, whose absolute values were very small. This limited variation amplifies the impact of noise and may degrade model performance. To mitigate this effect, target variables were standardized during the training of machine learning regression models, thereby improving model robustness. Finally, grid search was employed to optimize model parameters and reduce the risk of overfitting.

The final regression results are presented in **Table 2**, highlighting the predictive coefficient of determination (R²p), root mean square error of prediction (RMSEP), and residual predictive deviation (RPD), which collectively evaluate the model’s performance on the independent prediction set. Among the models tested, the PLSR model demonstrated the best overall performance in this study. Using the reconstructed spectral data, the D1 preprocessing method achieved the highest R²p value of 0.8523 for nitrogen prediction. For phosphorus prediction, the MSC preprocessing method yielded the best performance with an R²p of 0.7022. In the case of potassium prediction, the MSC method also achieved the highest R²p value of 0.8087. In this study, PLSR was selected as a primary regression model due to its inherent advantages in handling high-dimensional, collinear spectral data. Compared with Support Vector Regression (SVR) and Random Forest (RF), PLSR exhibits superior robustness and stability under limited sample conditions, while simultaneously integrating dimensionality reduction and regression. Its minimal parameter tuning requirements and strong interpretability—through regression coefficients and VIP scores—further enhance its suitability for hyperspectral analysis. These characteristics make PLSR a particularly effective and widely adopted approach in spectroscopic modeling for nutrient estimation tasks.

**Table 2:** Comparison of prediction results of ground truth spectra and reconstructed spectra on three nutrient components in the prediction set.

Nutrient	Spetra	Preprocessing method+Model	R²p	RMSEp	RPD	Spetra	Preprocessing method+Model	R²p	RMSEp	RPD
Nitrogen	Ground Truth	RAW+PLSR	0.8396	0.3920	2.4968	Reconstruct	RAW+PLSR	0.8322	0.4765	2.4411
		RAW+SVR	0.8203	0.5081	2.3589		RAW+SVR	0.3200	1.0500	1.2126
		RAW+RF	0.6558	0.7032	1.7045		RAW+RF	0.5656	0.7899	1.5173
		MSC+PLSR	0.8593	0.4495	2.6661		MSC++PLSR	0.8519	0.4121	2.5991
		MSC+SVR	0.6880	0.6695	1.7903		MSC+SVR	0.7379	0.5483	1.9532
		MSC+RF	0.6936	0.6634	1.8066		MSC+RF	0.6979	0.6588	1.8194
		D1+PLSR	0.8738	0.4850	2.6024		**D1+PLSR**	**0.8523**	**0.4850**	**2.6024**
		D1+SVR	0.8434	0.4742	2.5273		D1+SVR	0.6478	0.5252	1.6849
		D1+RF	0.7925	0.5460	2.1952		D1+RF	0.7267	0.6266	1.9128
		**D1+MSC+PLSR**	**0.9038**	**0.3035**	**3.2246**		D1+MSC+PLSR	0.8394	0.4662	2.4951
		D1+MSC+SVR	0.7768	0.4181	2.1168		D1+MSC+SVR	0.7146	0.6403	1.8719
		D1+MSC+RF	0.8072	0.52625	2.2775		D1+MSC+RF	0.7393	0.5939	1.9587
Phosphorus	Ground Truth	RAW+PLSR	0.4512	0.7719	1.3499	Reconstruct	RAW+PLSR	0.3918	0.8127	1.2822
		RAW+SVR	0.3671	0.7832	1.2570		RAW+SVR	0.2160	0.9227	1.1294
		RAW+RF	0.3189	0.8023	1.2117		RAW+RF	0.3062	0.8097	1.2006
		**MSC+PLSR**	**0.6950**	**0.5727**	**1.810**		**MSC+PLSR**	**0.7022**	**0.5658**	**1.8326**
		MSC+SVR	0.3532	0.7817	1.2434		MSC+SVR	0.3947	0.7659	1.2854
		MSC+RF	0.4200	0.7403	1.3131		MSC+RF	0.5423	0.7016	1.4782
		D1+PLSR	0.6471	0.6160	1.6835		D1+PLSR	0.6232	0.6365	1.6293
		D1+SVR	0.4070	0.7582	1.2986		D1+SVR	0.3918	0.6858	1.2823
		D1+RF	0.3864	0.7712	1.2766		D1+RF	0.3934	0.8077	1.2839
		D1+MSC+PLSR	0.5880	0.6656	1.5580		D1+MSC+PLSR	0.5587	0.6889	1.5053
		D1+MSC+SVR	0.4151	0.7529	1.3075		D1+MSC+SVR	0.4834	0.5464	1.3913
		D1+MSC+RF	0.4283	0.7841	1.3226		D1+MSC+RF	0.5249	0.7148	1.4508
Potassium	Ground Truth	RAW+PLSR	0.6684	0.6667	1.7367	Reconstruct	RAW+PLSR	0.7236	0.4805	1.9021
		RAW+SVR	0.5801	0.7502	1.5433		RAW+SVR	0.4942	0.6371	1.4062
		RAW+RF	0.5177	0.8040	1.4400		RAW+RF	0.4249	0.8780	1.3187
		MSC+PLSR	0.7350	0.6592	1.9425		**MSC+PLSR**	**0.8087**	**0.3997**	**2.2866**
		MSC+SVR	0.5181	0.6836	1.4406		MSC+SVR	0.4369	0.8688	1.3326
		MSC+RF	0.6254	0.7836	1.6340		MSC+RF	0.5283	0.5283	1.4560
		D1+PLSR	0.8182	0.5458	2.3458		D1+PLSR	0.7293	0.6661	1.9221
		D1+SVR	0.5244	0.7984	1.4501		D1+SVR	0.5283	0.7952	1.4560
		D1+RF	0.5065	0.8133	1.4235		D1+RF	0.5800	0.7503	1.5432
		**D1+MSC+PLSR**	**0.8370**	**0.5170**	**2.4767**		D1+MSC+PLSR	0.7225	0.5633	1.8986
		D1+MSC+SVR	0.4721	0.8412	1.3763		D1+MSC+SVR	0.4831	0.6571	1.3909
		D1+MSC+RF	0.6300	0.7788	1.6440		D1+MSC+RF	0.5542	0.6102	1.4977

The values shown in bold represent the highest (or best-performing) results among the compared methods in each experiment.

In related studies, Li used hyperspectral data in the range of 1,100–2,500 nm with a spectral resolution of 8 nm (totaling 175 wavelengths) to determine nitrogen concentrations in different tissues of slash pine, yielding a coefficient of determination (R²cv) of 0.66 in cross-validation (Li et al., 2022). Shu Meiyan et al. employed spectral decomposition methods to estimate the nitrogen status of maize leaves and identified sensitive wavelengths at 470 nm, 538 nm, 638 nm, 682 nm, 710 nm, 734 nm, and 830 nm. Using a CARS-SVR regression model, they achieved a R² of 0.68 on the test set (Meiyan et al., 2023). In another study, Yakun Zhang conducted *in situ*, non-destructive detection of nitrogen content in soybean leaves using hyperspectral imaging in the 380–1000 nm range. The model attained a maximum R²p of 0.9428 on the validation set. However, it is noteworthy that the nitrogen content range in that study was relatively wide (8.275–44.724 mg/g), and the hyperspectral data included 707 spectral bands—factors that likely contributed to the model’s high performance (Zhang et al., 2024). Similarly, Di Lin et al. used *in situ* hyperspectral data (400–900 nm) to predict nitrogen, phosphorus, and potassium content in the maize canopy. Their model achieved validation R² values of 0.821, 0.732, and 0.773 for nitrogen, phosphorus, and potassium, respectively (Lin et al., 2024). These results are comparable to those obtained in the present study. Considering that the hyperspectral data used in this research were reconstructed rather than directly acquired, the findings strongly demonstrate the effectiveness and reliability of the proposed methodology.

**Figure 10:**
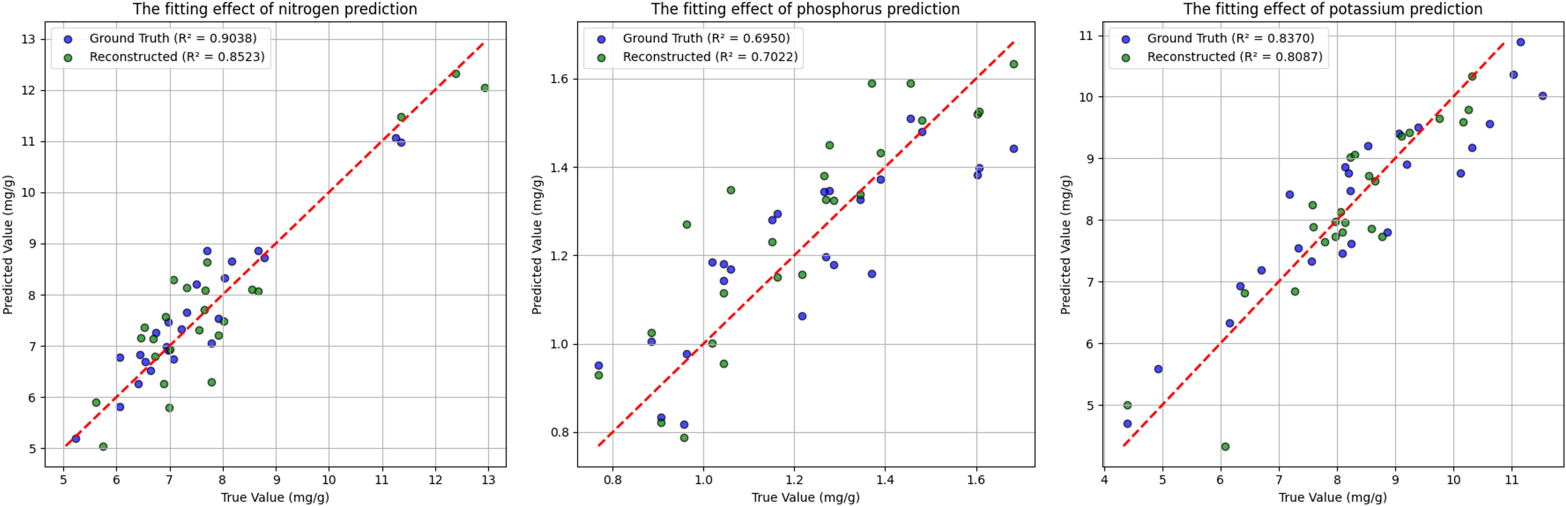
Comparison of scatter plot fitting results of three nutrients obtained from ground truth spectra and reconstructed spectra on the test set.

Finally, each element was evaluated using its respective optimal model on both ground-truth and reconstructed test sets, and the resulting regression performances are illustrated in **Figure 10**. The figure highlights the differences in the R²p values among the three elements. It is noteworthy that in this study, the prediction performance for phosphorus based on reconstructed spectra was slightly superior to that achieved using ground-truth hyperspectral data. This phenomenon may be primarily attributed to the inherently low phosphorus concentration—typically ranging between 0.8 mg/g and 1.6 mg/g—as well as its distribution across both needle and root tissues. Compared to nitrogen and potassium, phosphorus exhibits a more dispersed spatial distribution, which increases the uncertainty in machine learning-based regression. Nevertheless, this outcome indirectly supports the reliability and robustness of our reconstructed spectral data.

## 4 Discussion

### 4.1 Comparison of prediction performance between the original model and the improved model

As detailed in Section 2.2, the original MIRNet model reconstructed hyperspectral images within the 400–700 nm range, encompassing 31 spectral bands. In this study, we extended the spectral range to 400–1000 nm by utilizing 176 bands, thereby incorporating the near-infrared (NIR) region. While most existing research and publicly available datasets on hyperspectral reconstruction focus primarily on the visible spectrum (<700 nm), many hyperspectral imaging studies have already expanded into the NIR or even infrared regions. As a result, although hyperspectral reconstruction approaches offer cost advantages, their performance often lags behind that of direct hyperspectral imaging, particularly in the extended spectral range. Despite the inherent challenges of reconstructing NIR spectra, our approach achieved low-error reconstruction across the full 400–1000 nm spectrum.

For comparative analysis, we applied the original MIRNet model to RGB images of pine canopy needles to reconstruct hyperspectral images across 31 bands (400–700 nm), as summarized in **Table 3**. The reconstructed spectral data were then used for nutrient content prediction to evaluate the model’s effectiveness. Spectral preprocessing and feature band selection methods were kept consistent. The results showed that the highest R² values on the prediction set for nitrogen, phosphorus, and potassium were 0.8159, 0.5040, and 0.7007, respectively—representing decreases of 4.3%, 28.2%, and 13.4% compared to the improved reconstruction model. These findings emphasize the importance of both enhancing the reconstruction model and expanding the spectral range.

**Table 3:** Comparison of prediction results between improved model and original model (Only display the best results).

Nutrient	Model	Preprocessing method+Model	R²p	RMSEp	RPD	Model	Preprocessing method+Model	R²p	RMSEp	RPD
nitrogen	Improved	**D1+PLSR**	**0.8523**	**0.4850**	**2.6024**	Original	MSC+PLSR	0.8159	0.4991	2.3308
phosphorus	Improved	**MSC+PLSR**	**0.7022**	**0.5658**	**1.8326**	Original	D1+PLSR	0.5040	0.6934	1.4199
potassium	Improved	**MSC+PLSR**	**0.8087**	**0.3997**	**2.2866**	Original	D1+PLSR	0.7007	0.6333	1.8280

The values shown in bold represent the highest (or best-performing) results among the compared methods in each experiment.

### 4.2 The influence of different degrees of fuzziness on the accuracy of model reconstruction

**Figure 11:**
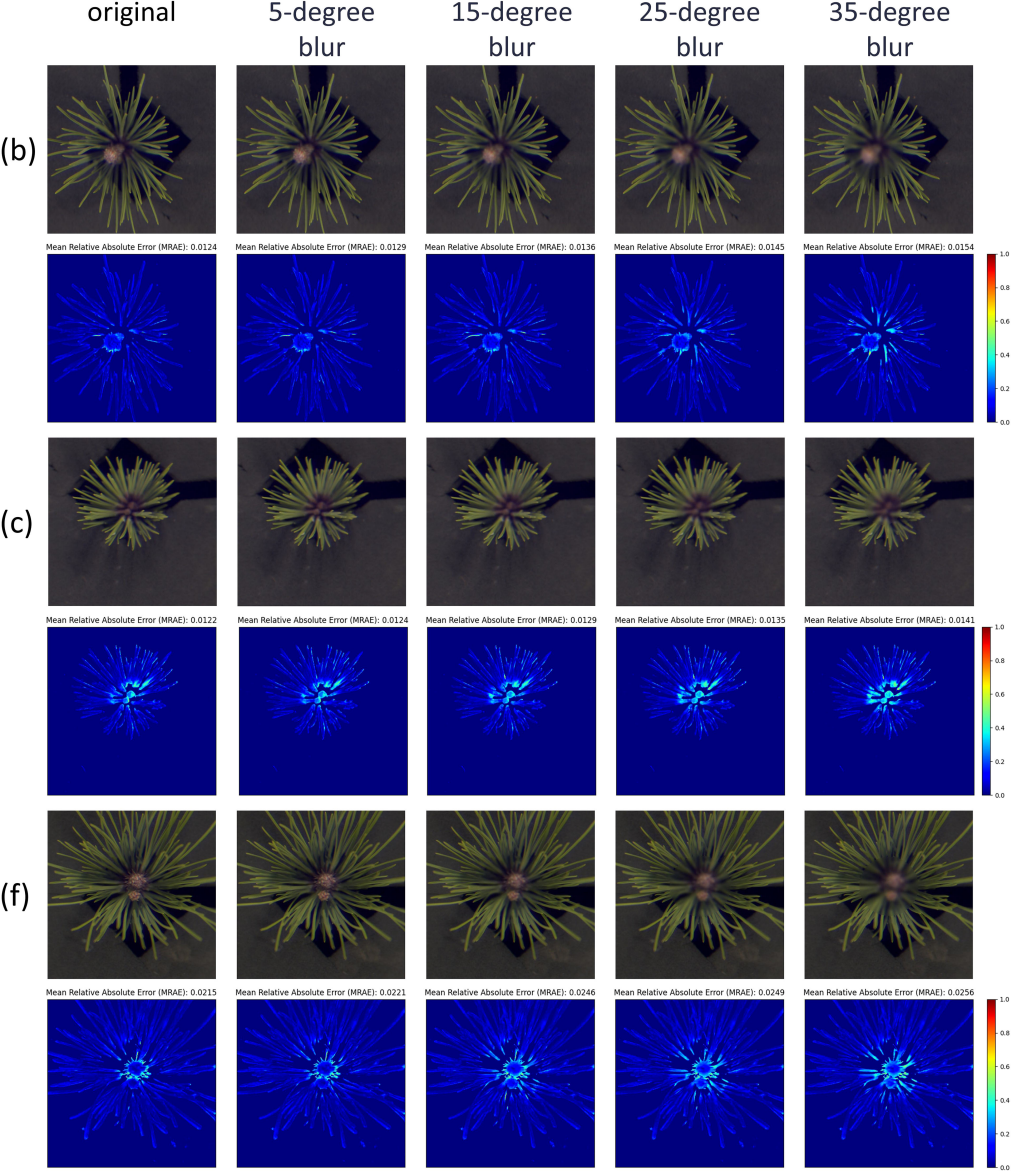
The reconstruction results under different degrees of Gaussian blur. From left to right: the original image, and Gaussian blur kernel sizes of 5, 15, 25, and 35. The results are shown for three representative samples: **(b, c, f)** from the eight samples in **Figure 6**.

In Section 3.3, we observed that nearly all samples exhibited increased reconstruction errors in the central region at the 900 nm band. This phenomenon is likely due to image blurring caused by depth-of-field effects during image acquisition. To test this hypothesis, we applied Gaussian blurring with varying kernel sizes to the central regions of samples b, c, and f, where the issue was most pronounced. These blurred images were then used for hyperspectral reconstruction, and the resulting errors at 900 nm were visualized using heatmaps, as shown in **Figure 11**. From left to right, the images represent the original sample and those blurred with Gaussian kernels of sizes 5, 15, 25, and 35, respectively. The heatmaps clearly indicate that as blurring intensity increases, reconstruction errors in the central region become more severe. The average MARE for the three samples increased from 0.0124, 0.0122, and 0.0215 to 0.0154, 0.0141, and 0.0256, respectively, supporting our hypothesis.

**Figure 12:**
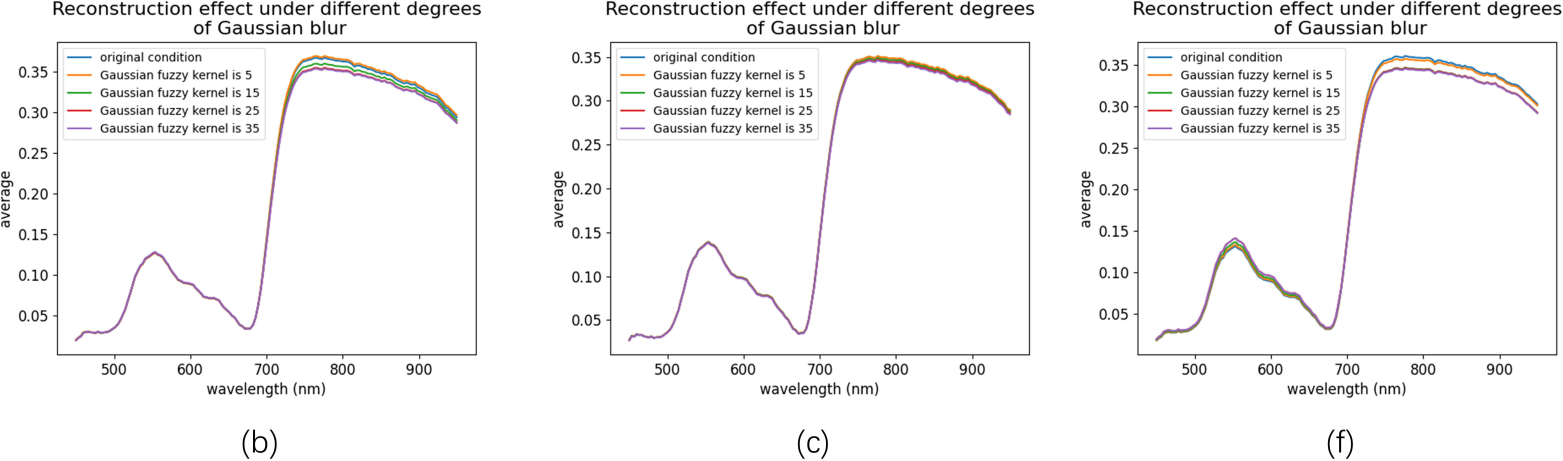
Spectral curves reconstructed under different degrees of Gaussian blur. The curves are shown for three representative samples: **(b, c, f)** from the eight samples in **Figure 6**, under varying Gaussian blur kernel sizes (5, 15, 25, and 35).

The reconstructed spectral reflectance curves in **Figure 12** further demonstrate the impact of blurring. For samples b and c, the spectral range from 450 to 750 nm remained largely unaffected. However, beyond 750 nm, sample b exhibited greater sensitivity to blurring than sample c. When the Gaussian kernel size exceeded 5, the reconstructed spectra for sample b consistently fell below the original values, whereas sample c maintained stable spectra across all levels of blurring. This robustness is likely attributed to the smaller surface area of sample c.

In contrast, sample f showed significant sensitivity to blurring in the 500–600 nm and 750–950 nm ranges. Notably, reconstruction errors increased markedly when Gaussian kernels of sizes 5 and 15 were applied. However, with larger kernel sizes, the errors in both spectral ranges gradually stabilized. Importantly, for all three samples, reconstruction errors remained nearly unchanged when Gaussian kernel sizes reached 25 and 35, demonstrating the MIRNet model’s remarkable robustness in handling highly blurred images.

Given the structural characteristics of pine needle canopies, depth-of-field effects during image acquisition are unavoidable. However, this limitation also presents valuable insights for future research. Since the ultimate goal of this study is to deploy the model on external agricultural equipment, real-world image captures will inevitably involve variations in equipment, camera angles, and lighting, leading to varying degrees of image blurring. Therefore, future research will focus on integrating spatial and spectral super-resolution techniques to enhance the quality of original RGB images, thereby improving the overall accuracy of hyperspectral reconstruction.

## 5 Conclusions

This study investigates the application of hyperspectral image reconstruction techniques for estimating nutrient content in pine canopy needles, with the goal of enhancing model performance for practical deployment in forestry and agriculture. We improved the architectures of MIRNet, HRNet, MPRNet, and Restormer to reconstruct hyperspectral images from three-channel RGB inputs, extending the spectral range to 400–1000 nm across 176 bands to meet the spectral requirements for predicting three key nutrients. All four networks demonstrated strong reconstruction performance; however, MIRNet consistently outperformed the others across both the 400–700 nm and 700–1000 nm ranges, despite having the fewest parameters and lowest computational complexity. Owing to its superior accuracy and lightweight design, MIRNet was selected as the reconstruction model for subsequent regression experiments.

For nutrient content prediction, we employed the hyperspectral data reconstructed by MIRNet and applied a partial least squares regression (PLSR) model to estimate nitrogen (N), phosphorus (P), and potassium (K) concentrations in the samples. The predictions on the test set closely aligned with those derived from the original hyperspectral dataset captured by a hyperspectral camera. Additionally, we conducted the same experiment using the original, unmodified network architectures, which reconstructed hyperspectral data with only 31 bands. The prediction accuracy for all three nutrients was significantly lower than that achieved using our enhanced model, underscoring the value of both architectural improvements and spectral range expansion.

Overall, these findings demonstrate the effectiveness of our approach in accurately reconstructing hyperspectral information and predicting plant nutrient content. By enabling *in-situ* estimation through RGB-based hyperspectral reconstruction, the proposed method substantially reduces system complexity and cost while maintaining high predictive performance. This research provides both a solid theoretical foundation and practical advancements for precision forestry, supporting enhanced plant management, optimized resource allocation, and improved productivity under real-world field conditions.

## Data Availability

The original contributions presented in the study are included in the article/supplementary material. Further inquiries can be directed to the corresponding authors.
